# Evidence that talin alternative splice variants from *Ciona intestinalis *have different roles in cell adhesion

**DOI:** 10.1186/1471-2121-7-40

**Published:** 2006-12-06

**Authors:** Richard H Singiser, Richard O McCann

**Affiliations:** 1 University of Kentucky College of Medicine, Department of Molecular and Cellular Biochemistry, 741 South Limestone Street, Lexington, KY 40536, USA

## Abstract

**Background:**

Talins are large, modular cytoskeletal proteins found in animals and amoebozoans such as *Dictyostelium discoideum*. Since the identification of a second talin gene in vertebrates, it has become increasingly clear that vertebrate Talin1 and Talin2 have non-redundant roles as essential links between integrins and the actin cytoskeleton in distinct plasma membrane-associated adhesion complexes. The conserved C-terminal I/LWEQ module is important for talin function. This structural element mediates the interaction of talins with F-actin. The I/LWEQ module also targets mammalian Talin1 to focal adhesion complexes, which are dynamic multicomponent assemblies required for cell adhesion and cell motility. Although Talin1 is essential for focal adhesion function, Talin2 is not targeted to focal adhesions. The nonvertebrate chordate *Ciona intestinalis *has only one talin gene, but alternative splicing of the talin mRNA produces two proteins with different C-terminal I/LWEQ modules. Thus, *C. intestinalis *contains two talins, Talin-a and Talin-b, with potentially different activities, despite having only one talin gene.

**Results:**

We show here that, based on their distribution in cDNA libraries, Talin-a and Talin-b are differentially expressed during *C. intestinalis *development. The I/LWEQ modules of the two proteins also have different affinities for F-actin. Consistent with the hypothesis that Talin-a and Talin-b have different roles in cell adhesion, the distinct I/LWEQ modules of Talin-a and Talin-b possess different subcellular targeting determinants. The I/LWEQ module of Talin-a is targeted to focal adhesions, where it most likely serves as the link between integrin and the actin cytoskeleton. The Talin-b I/LWEQ module is not targeted to focal adhesions, but instead preferentially labels F-actin stress fibers. These different properties of *C. intestinalis *the Talin-a and Talin-b I/LWEQ modules mimic the differences between mammalian Talin1 and Talin2.

**Conclusion:**

Vertebrates and *D. discoideum *contain two talin genes that encode proteins with different functions. The urochordate *C. intestinalis *has a single talin gene but produces two separate talins by alternative splicing that vary in a domain crucial for talin function. This suggests that multicellular organisms require multiple talins as components of adhesion complexes. In *C. intestinalis*, alternative splicing, rather than gene duplication followed by neo-functionalization, accounts for the presence of multiple talins with different properties. Given that *C. intestinalis *is an excellent model system for chordate biology, the study of Talin-a and Talin-b will lead to a deeper understanding of cell adhesion in the chordate lineage and how talin functions have been parceled out to multiple proteins during metazoan evolution.

## Background

**Figure 1:**
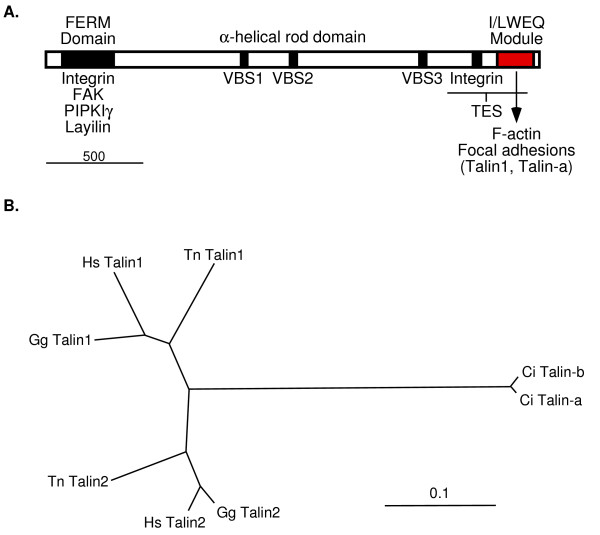
**A. Talin structure**. The modular structure of talin is illustrated and representative binding sites for partners of vertebrate Talin1 are indicated [1, 2, 13]. All known talins, including *C. intestinalis *talin, contain an N-terminal FERM domain and a C-terminal I/LWEQ module [18]. The FERM domain is a conserved element that links various proteins to the plasma membrane [38]. The I/LWEQ module is a conserved F-actin-binding element that also targets Talin1 to focal adhesions in mammalian cells [13]. We show in this report that the I/LWEQ module of *C. intestinalis *Talin-a also contains a focal adhesion targeting signal. VBS: vinculin binding sites of Talin1. Scale bar: 500 amino acids. **B. **Unrooted tree showing the phylogenetic relationships of full-length talins from the mammal *Homo sapiens *(Hs), the bird *Gallus gallus *(Gg), the pufferfish *Tetraodon nigroviridis *(Tn), and *Ciona intestinalis *(Ci). The vertebrate Talin1 and Talin2 form orthologous groups, with *C. intestinalis *Talin-a/b as the outgroup. The complete sequence alignment upon which this tree is based and an identity/similarity matrix for these talins are shown in Additional File 1. Human Talin1 and *C. intestinalis *Talin-b are 56% identical and 69.4% similar over 2541 amino acids. Scale bar: 10% sequence divergence.

The amoebozoan and animal talins (Fig. 1) are an ancient family of modular proteins that link the actin cytoskeleton to the plasma membrane in several different multicomponent adhesion complexes [1-3]. The amoebozoan cellular slime mold *Dictyostelium discoideum *contains two talins, each of which has a different role in the *D. discoideum *life cycle. TalA is required for cell motility and adhesion to the substrate in undifferentiated amoebae [4,5], and TalB is required for morphogenesis during multicellular differentiation [6,7]. Vertebrates also have two talin genes, *TLN1 *and *TLN2*, which encode Talin1 and Talin2, respectively [3,8-10]. Talin1 is required for cell adhesion and motility and is the primary talin component of focal adhesions [6,7,11]. This role for Talin1 is analogous to that of TalA in adhesion and migration of *D. discoideum *amoebae [4,5]. Although Talin2 has not been completely characterized, the available evidence indicates that Talin1 and Talin2 have non-redundant roles in vertebrates, with Talin2 serving as a component of stable adhesions in differentiated tissues such as striated muscle [10]. This suggests that Talin2 may be an analog of *D. discoideum *TalB, which is required for multicellular differentiation [6,7]. Thus, at opposite ends of the large phylogenetic distance that separates amoebozoans and vertebrates within the opisthokont (i.e., animals, amoebozoans, and fungi) lineage [3,12], two different talins are required for cell adhesion in distinct adhesion assemblies.

In contrast to *D. discoideum *and vertebrates, invertebrates including *Drosophila melanogaster *and *Caenorhabditis elegans *and the nonvertebrate chordate *Ciona intestinalis *have only one talin gene [3]. However, alternative splicing of the talin mRNA in *D. melanogaster *and *C. intestinalis *produces multiple talins that vary at their C-termini [3]. Therefore, despite having only one talin gene, multiple talin proteins can account for the biological functions of talin in these organisms.

Little is currently known about the functions of these talin splice variants. In this report we have determined that the I/LWEQ modules of the alternative splice variants of *C. intestinalis *talin have different actin-binding affinities and different subcellular targeting signals. We show that the Talin-b I/LWEQ module has a higher affinity for F-actin than the Talin-a I/LWEQ module and is also targeted to actin stress fibers in cells. In contrast, the Talin-a I/LWEQ module does not label stress fibers, but is instead targeted to focal adhesions. These differences are similar to those seen with Talin1 and Talin2 in vertebrates, where Talin1 is the link between integrins and the actin cytoskeleton in focal adhesions [11,13]. These results suggest that alternative splicing is an effective mechanism for producing distinct talin proteins with different functions in *C. intestinalis*. The recent completion of its genome sequence has increased the utility of *C. intestinalis *as a model chordate [14-17]. Thus, further studies of Talin-a and Talin-b in *C. intestinalis *will be useful in identifying fundamental roles for distinct talin proteins in cell adhesion in chordates.

## Results

### Identification of *C. intestinalis *Talin-a and Talin-b

During a previous analysis of the evolutionary history of amoebozoan and animal talins we determined that the *C. intestinalis *genome contains one talin gene, but that alternative splicing produces two talin proteins, based on the presence of multiple, distinct cDNA sequences in the GenBank expressed sequence tag (EST) database [3]. Like all the other talins previously identified, the full-length *C. intestinalis *talin contains an N-terminal FERM domain linked to a C-terminal I/LWEQ module by a central α-helical rod domain [18]. In the present study we have identified multiple cDNA clones corresponding to *C. intestinalis *Talin-a and Talin-b (Table 1). These data indicate that Talin-a is perhaps more widely expressed, based on the distribution of cDNAs in the available libraries, than Talin-b. Talin-a cDNAs were found in libraries from the whole adult organism, heart, gastrula/neurula, tailbud embryo, free swimming larva, blood cells, neural complex, and testis. In addition to these *C. intestinalis *Talin-a cDNAs, several Talin-a ESTs were also identified from the tunicate *Molgula tectiformis*. Talin-b cDNAs have been identified from libraries prepared from blood cells and the adult organism. We have also identified a Talin-b EST from a cDNA library prepared from gastrula of the tunicate *Ciona savignyi*, so this splice variant is not restricted only to *C. intestinalis *(Table 1).

**Table 1:** Distribution of Talin-a and Talin-b cDNAs.

**Talin-a**		**Talin-b**	
**GenBank Accession**	**Source**	**GenBank Accession**	**Source**

BW500911	adult	BW056464	blood cells
BW312515	heart	BW050858	blood cells
BW261786	gastrula/neurula	BW029542	blood cells
BW250883	tailbud embryo	BW029446	blood cells
BW229400	larva	BW029292	blood cells
BW054944	blood cells	BW050224	blood cells
BW170650	neural complex	BW482633	adult
BP020498	testis	BW522112	gastrula (*C. savignyi*)
CJ430072	larva (*M. tectiformis*)		

**Figure 2:**
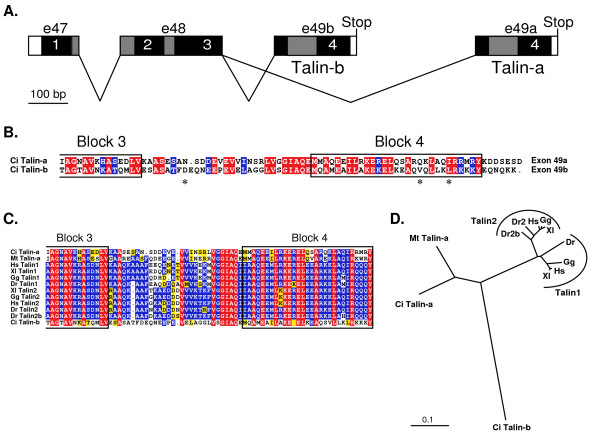
**A. *C. intestinalis *alternative splice variants Talin-a and Talin-b**. Exons 47, 48 and 49 encode the C-terminal I/LWEQ module of talin, which is composed of four conserved blocks (black boxes, 1–4 [2]). The alternative exon 49 specifies the 78 C-terminal amino acids of either Talin-a (e49a) or Talin-b (e49b). **B. Sequence comparison of Talin-a and Talin-b. **The alternative splice site is near the C-terminal boundary of Block 3. The asterisks identify three sequence polymorphisms (E D, S V, and I L) that were found in cDNAs isolated from two separate first-strand cDNA syntheses using total RNA from organisms from the Atlantic population of *C. intestinalis*, which is isolated from the Pacific population that was used to determine the genome sequence. Red, identical amino acids; blue, conserved changes. **C. Multiple sequence alignment of representative chordate talin C-termini. **The unique Talin-a and Talin-b sequences, through the final residue of Block 4, were aligned with 10 other chordate talins using CLUSTAL W [19]. Complete description of these sequences is available in reference [3]. Red, identical amino acids; blue, conserved; yellow, similar. **D. Chordate I/LWEQ module phylogenetic tree. **The guide tree from the alignment of the C-termini of chordate I/LWEQ modules in (**C**) was used to calculate this unrooted tree [20], which shows both the conservation and divergence of the C-termini of the chordate talins. Vertebrate Talin1 and Talin2 are clustered in two orthologous branches. *C. intestinalis *Talin-a and the Talin-a ortholog from the tunicate *Molgula tectiformis *also group together. The sequence divergence of *C. intestinalis *Talin-b from the other talins is illustrated by the long single branch. Species: Ci, *C. intestinalis*; Mt, *Molgula tectiformis*; Hs, *Homo sapiens*; Xl, *Xenopus laevis*; Gg, *Gallus gallus *(chicken); Dr, *Danio rerio *(zebrafish). Scale bar: 10% sequence divergence.

Unambiguous Talin-a and Talin-b cDNAs were identified by comparison of the C-terminal protein sequences (Fig. 2B) with the original Talin-a and Talin-b that we have previously identified [3]. CJ430072 is an EST for Talin-a from a larva cDNA library of the tunicate *Molgula tectiformis*; BW522112 is an EST for Talin-b from a gastrula library from *Ciona savignyi*. All of the other sequences are from *C. intestinalis*. Talin-a and Talin-b alignments of the *C. intestinalis *protein sequences derived from these ESTs are shown in Additional File 2 and Additional File 3, respectively.

Comparison of the variant cDNA sequences of *C. intestinalis *with the genomic DNA sequence showed that the two cDNAs are the result of alternative splicing at the end of exon 48 of the talin transcript (Fig. 2A). The alternative final protein-coding exon 49 produces talin proteins that are 42% identical over their C-terminal 78 amino acids (Fig. 2B). This sequence divergence is greater than that between the paralogous human Talin1 and zebrafish Talin2, which are 82% identical over the same region, and is more comparable to the divergence between human Talin1 and *Caenorhabditis elegans *talin, which are 57% identical at their C-termini. Multiple sequence alignment (Fig. 2C) of the C-termini of representative chordate talins using CLUSTAL W [19] and conversion of that alignment into an unrooted tree (Fig. 2D) using TreeView [20] illustrate the relatively high level of divergence of the C-terminus of the I/LWEQ module of *C. intestinalis *Talin-b from the other chordate talins.

### Actin binding activity of the Talin-a and Talin-b I/LWEQ modules

**Figure 3:**
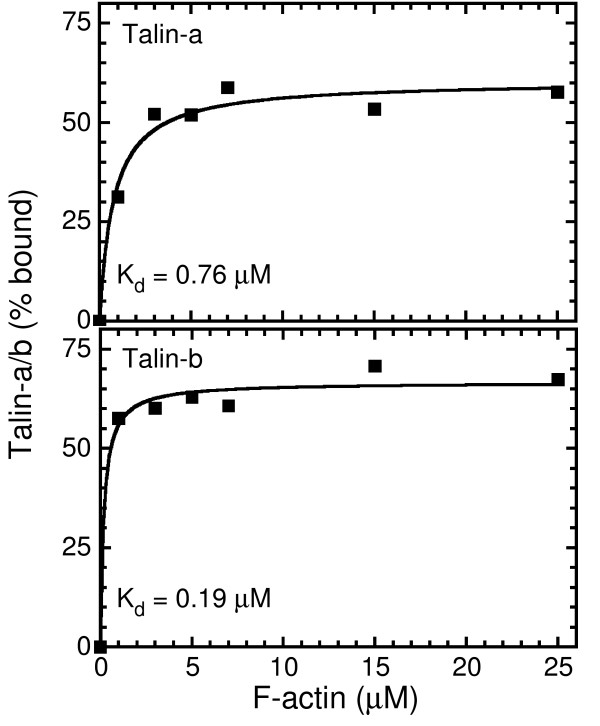
**Actin binding of Talin-a and Talin-b I/LWEQ modules**. Actin binding curves for the interaction of GST-Talin-a.2341–2531 and GST-Talin-b.2341–2531 with F-actin were calculated with GraphPad Prism 4.0 as previously described [22]. The Talin-b I/LWEQ module bound to F-actin with a 4-fold higher affinity than that of Talin-a (K_d _= 0.19 μ*M *vs. 0.76 μ*M*). Each data point is the average of three independent measurements.

The I/LWEQ module of talins and other members of the superfamily (e.g., fungal Sla2 and animal Hip1) specifies actin binding in these proteins [2,9,21]. We have recently determined that the C-terminus of the I/LWEQ module, corresponding to the splice variant sequences of *C. intestinalis *Talin-a and Talin-b, contains the minimal actin-binding site of mammalian Talin1 [13]. This observation led us to determine whether the actin-binding affinities of the I/LWEQ modules of Talin-a and Talin-b for F-actin are different (Fig. 3). We found that the Talin-b construct binds to F-actin with an apparent K_d _of 0.19 μ*M*, while the Talin-a I/LWEQ module bound with an apparent K_d _of 0.76 μ*M*. These values are similar to those we previously determined for mammalian Talin1 and Talin2, whose K_d _values were 0.31 μ*M *and 0.88 μ*M*, respectively [22].

### Subcellular targeting of Talin-a and Talin-b

**Figure 4:**
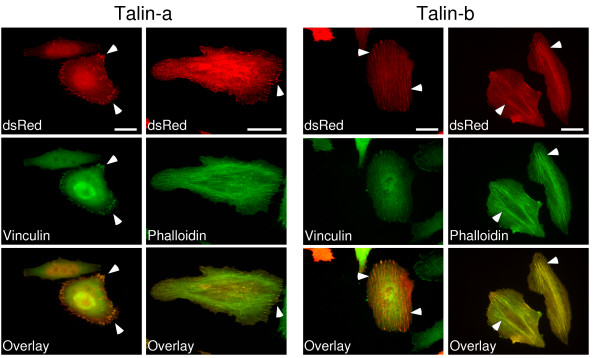
**Subcellular targeting of the I/LWEQ modules of Talin-a and Talin-b**. HeLa cells were transiently transfected with either the dsRed-Talin-a.2341–2531 or the dsRed-Talin-b.2341–2531 fusion construct (I/LWEQ module) and counterstained with vinculin to independently label focal adhesions or fluorescein-phalloidin to label F-actin, as previously described [13]. The Talin-a I/LWEQ module targeted to focal adhesions, where the fluorescence signals for dsRed-Talin-a.2341–2531 and the focal adhesion component vinculin overlap (Column 1, overlay). The Talin-a I/LWEQ module did not preferentially localize to actin stress fibers (Column 2). In contrast to Talin-a, the Talin-b I/LWEQ module was not targeted to focal adhesions (Column 3). However, dsRed-Talin-b.2341–2531 did colocalize with actin stress fibers, as shown by the colocalization of the fluorescence signals of dsRed-Talin-b.2341–2531 with phalloidin-stained F-actin (Column 4, overlay).

Focal adhesions are dynamic multi-component adhesion complexes required for cell adhesion and cell motility [23,24]. Talin1 is required for the assembly of focal adhesions [25], and we recently determined that the I/LWEQ module of Talin1 is also essential for targeting this talin isoform to focal adhesions in mammalian cells [13]. Because *C. intestinalis *Talin-a and Talin-b vary in this subcellular targeting region of talin, we used expression of dsRed-Talin-a and dsRed-Talin-b fusion proteins to determine whether the Talin-a and Talin-b I/LWEQ modules also have different subcellular targeting determinants in mammalian cells. Interestingly, we found that the I/LWEQ module of Talin-a strongly labels focal adhesions in HeLa cells (Fig. 4, column 1, arrowheads), where the dsRed-Talin-a fusion protein colocalized with the diagnostic focal adhesion component vinculin (overlay). Although the Talin-a I/LWEQ module does interact with F-actin *in vitro *(Fig. 3), it did not preferentially label actin stress fibers in cells (Fig. 4 column 2; arrowheads identify dsRed-Talin-a in focal adhesions at the ends of stress fibers). In contrast to Talin-a, the Talin-b I/LWEQ module was not targeted to focal adhesions (Fig. 4, column 3; arrowheads identify stress fiber localization of dsRed-Talin-b I/LWEQ module), but instead strongly labeled actin stress fibers in HeLa cells (Fig. 4, column 4, arrowheads), where dsRed-Talin-b I/LWEQ module colocalized with FITC-phalloidin-labeled F-actin (overlay).

## Discussion

Analysis of EST data produced primarily by Nori Satoh and colleagues of Kyoto University [26] has shown that alternative splicing of talin pre-mRNA leads to the production of two proteins with different C-termini in *C. intestinalis*. The variable distributions of Talin-a and Talin-b in different cDNA libraries suggest that Talin-a and Talin-b are differentially expressed during *C. intestinalis *development. We have also shown that the variant I/LWEQ modules of Talin-a and Talin-b have different biological properties: Talin-b has the higher affinity for F-actin and preferentially labels actin stress fibers, while Talin-a is targeted to focal adhesions. Therefore, alternative processing is a mechanism for producing multiple talin proteins, with distinct activities associated with a critical structural element, in these nonvertebrate chordates, even though the *C. intestinalis *genome contains only one talin gene.

Alternative splicing is a widespread mechanism for expanding the proteome. In humans, 30–65% of all gene products are subject to alternative splicing, and similar levels are also seen in other organisms, including the mouse, fruitfly, and nematode [27]. Alternative splicing frequently results in changes in protein structure that alter protein-protein interactions. For example, alternative splicing of the non-erythroid protein 4.1R produces variants that alter the efficiency of the interaction with the spectrin/actin complex by a factor of two [28]. This is similar to differences we have identified in the *C. intestinalis *talin alternative splice variants, where the Talin-b I/LWEQ module interacts with F-actin with a four-fold higher affinity than that of Talin-a. The higher affinity for F-actin may explain the preferential localization of the Talin-b I/LWEQ module to actin stress fibers in cells.

Alternative splicing can also alter subcellular targeting determinants of the resultant proteins [27]. HeLa cells represent a heterologous system for the study of tunicate talins *in vivo*, but we have recently used them to determine that the C-terminal I/LWEQ module of Talin1 contains a focal adhesion targeting determinant [13]. We have shown here that the Talin-a I/LWEQ module, but not that of Talin-b, is targeted to focal adhesions in HeLa cells. Although focal adhesions have not yet been directly observed in *C. intestinalis *cells, the *C. intestinalis *genome contains genes for several characteristic focal adhesion components that were previously thought to be restricted to vertebrates. These include at least 14 α-integrins, 5 β-integrins, focal adhesion kinase, vinculin, and α-actinin [15]. Vinculin and β-integrin are well-characterized Talin1 binding partners. Given the presence of these classical focal adhesion proteins in *C. intestinalis*, our results suggest that Talin-a is likely to function similarly to mammalian Talin1 as an adhesion complex component during *C. intestinalis *development. The lack of a focal adhesion-targeting determinant in the C-terminus of Talin-b may indicate that this protein is a component of other adhesion complexes in the organism, as we have seen with mammalian Talin2. Talin2 is not targeted to focal adhesions in undifferentiated cells but is preferentially targeted to costameres and intercalated disks, which are stable adhesions in differentiated striated muscle [10].

The presence of two related, but ancient and highly divergent talins (30% identical) with different physiological roles in *D. discoideum *[4-6] supports the hypothesis that gene duplication, followed by neo-functionalization, produced multiple talins that are required for cell adhesion during cell differentiation [3,29]. A similar explanation may also account for the maintenance of Talin1 and Talin2 in vertebrates since duplication of the ancestral animal talin early in the chordate lineage, more than 450 million years ago [3]. In support of this hypothesis regarding vertebrate talins, several recent studies have shown that these distinct proteins indeed have different roles. Talin1 and Talin2 interact with different proteins [3,30,31] and have different affinities for their common partner F-actin [22]. Talin1 is required for mammalian embryogenesis, probably as an essential component of adhesion complexes required for cell motility during gastrulation [32]. Talin2 is unable to complement this lethal phenotype. We have recently shown that the adult tissue distributions of mammalian Talin1 and Talin2 also vary, with Talin1 being the more widely expressed isoform [10]. Talin2 is most abundant in brain and muscle. We have shown that Talin2 is induced during muscle differentiation along with other muscle-specific proteins such as archvillin, integrin-β1_D_, and metavinculin, which is an alternative splice variant of vinculin [10]. As expected, Talin1 is a component of focal adhesions in undifferentiated myoblasts, but Talin2 is not found in focal adhesions of these cells or other mammalian cells. Talin2 is instead a component of stable adhesion assemblies such as costameres and intercalated disks in mature striated muscle [10]. Taken together, these studies indicate that vertebrate Talin1 and Talin2 are differentially expressed during cell differentiation and organismal development and that they function as non-redundant components of distinct adhesion complexes in different cells and tissues. Thus, in both *D. discoideum *and vertebrates, multiple talins are involved in talin functions.

Development in *C. intestinalis *is a complex, multistage process. Following fertilization and subsequent embryogenesis, a free-swimming tadpole larva containing a notochord, nervous system, and musculature settles upon a solid substrate and metamorphoses into a sessile, filter-feeding organism. The adult contains organs common to other chordates, including a digestive system, heart, and nervous system [33]. *C. intestinalis *has only one talin gene, but the existence of the splice variants Talin-a and Talin-b, which vary in the important C-terminal I/LWEQ module [13], further supports the emerging paradigm that talin function is due to the actions of distinct proteins, which are expressed at different times and in different places during chordate development [3,10]. The results presented here suggest that alternative splicing is the means by which two talins with different roles in cell adhesion, and perhaps cell differentiation, are produced from one talin gene in *C. intestinalis*. Thus, this mechanism may bridge the one-talin bottleneck present in invertebrates between the multicellular amoebozoan *D. discoideum *[34] and vertebrates [3]. Further studies at the cellular and molecular levels will identify when and where Talin-a and Talin-b are produced in *C. intestinalis*, and how they contribute to the assembly and function of adhesion complexes during development of this model organism.

## Conclusion

Taken together with previous studies showing that talin function is due to the activities of distinct proteins, which are differentially expressed and targeted to different adhesion complexes in multicellular organisms, our results show that alternative splicing of the *C. intestinalis *talin mRNA produces two different proteins, Talin-a and Talin-b. These proteins vary in the C-terminal I/LWEQ module, which is critical for talin function. Moreover, these differences lead to different activities of the alternative splice variants. Our data suggest that *C. intestinalis *Talin-a is involved in cell adhesion and motility in assemblies that are similar to focal adhesions. Identification of the structural determinants responsible for the different properties of Talin-a and Talin-b will expand our knowledge of how different talins are involved in cell adhesion in chordates. Given that *C. intestinalis *has recently re-emerged as a model for the study of chordate development [33,35], further research comparing the roles of Talin-a and Talin-b during *C. intestinalis *development will also increase our knowledge of how talin functions have evolved during animal evolution.

## Methods

### Identification of Talin-a and Talin-b

Talin-a and Talin-b cDNAs were identified in BLAST searches [36] of the GenBank non-human, non-mouse EST database using the C-terminal 250 amino acid sequence of *Ciona intestinalis *talin [GenBank: AABS01000025] as the query sequence [3]. Unambiguous Talin-a and Talin-b cDNAs were identified by comparison of the C-terminal protein sequences with the original Talin-a and Talin-b that we identified previously [3]. Full-length and partial C-terminal chordate talin sequences were aligned with CLUSTAL W [19] using the European Bioinformatics Institute server as previously described [3]. Unrooted trees were then calculated from these alignments using TreeView [20]. The sequence alignments in Fig. 2 were prepared for display using MacBoxShade as previously described [3].

### PCR amplification of Talin-a/b from *Ciona intestinalis *and protein preparation

Whole *Ciona intestinalis *adults were obtained from the Marine Biological Laboratory (Woods Hole, MA). Organisms were frozen in liquid nitrogen, pulverized, and homogenized. RNA was extracted using RNAqueous-Midi (Ambion). First strand cDNA was synthesized using Superscript II reverse transcriptase (Invitrogen) with 1 μg total RNA and the oligo dT primer (5'-GACTCGAGTCGACATCGATTTTTTTTTTTTTTTTT-3'). Talin-a/b regions were then amplified by PCR using gene specific primers with the first-strand cDNA as a template: Talin-a/b sense, 5'-CCGGAATTCATTTTGGAAGCCGCAAAATCAATCGC-3' Talin-a antisense, 5'-CCTGGCGCGCCTTAATCGGATTCAGAATCATCCTTGT-3' Talin-b antisense, 5'-CCTGGCGCGCCCTATTTCTTTTGGTTTTGCTCGTATTTC-3' PCR amplicons were recovered using pCR2.1-TOPO (Invitrogen) and then subcloned into pET-41 (Novagen) for production of GST-fusion proteins. The proteins produced correspond to amino acids 2341–2531 for both Talin-a and Talin-b. All constructs were verified by DNA sequencing. Recombinant GST fusion proteins were prepared as previously described [2,22].

### F-Actin Binding

Actin was obtained from Cytoskeleton, Inc (Denver, CO). The binding of the I/LWEQ modules of Talin-a and Talin-b to F-actin was determined as previously described [2,22]. Briefly, F-actin co-sedimentation assays were performed in buffer A (2 m*M *Tris, pH 8.0, 0.2 m*M *CaCl_2_, 0.2 m*M *ATP, and 0.5 m*M *dithiothreitol), keeping the concentration of GST-Talin-a/b.2341–2531 constant at 4.0 μ*M *while increasing the actin concentration from 0 to 25 μ*M *in a total volume of 50 μl. Actin polymerization was induced by addition of 2 m*M *MgCl_2 _and 50 m*M *KCl and allowed to proceed for 60 minutes at 22°C. The critical concentration for actin polymerization is 0.71 μM under these conditions. Following centrifugation at 160000 *g *for 20 minutes at 22°C, the supernatant and pellet fractions were separated for subsequent analysis with SDS-PAGE using 12% gels. Proteins were stained with Coomassie Blue G250. Digital quantification of the cosedimentation data was performed using NIH Image v1.62 and binding curves were calculated using GraphPad Prism 4.0 as previously described [2,22]. The R^2 ^values for the binding curves were 0.95 for Talin-a and 0.99 for Talin-b. Neither GST-Talin-a.2341–2531 nor GST-Talin-b.2341–2531 sedimented at 160,000 *g *in the absence of F-actin. We have determined previously that the presence of the GST purification tag has no effect on actin binding of I/LWEQ module proteins [2,22]. The concentration of actin was determined using its molar extinction coefficient at 290 nm. The concentrations of GST-Talin-a and GST-Talin-b were determined using the BCA protein assay (Pierce) and by calculating the extinction coefficient of each protein [37].

### Immunofluorescence

*C. intestinalis *Talin-a/b I/LWEQ module constructs were subcloned from pET-41 to pDsRedC2-monomer (BD Biosciences) and verified by DNA sequencing. HeLa cells (American Type Culture Collection CCL-2) were maintained in Dulbecco's modified Eagle's medium (DMEM) supplemented with 10% FBS, 100 μg/ml penicillin, 100 μg/ml streptomycin and 10 μg/ml gentamicin. Glass coverslips were ethanol-washed and coated with 10 μg/ml fibronectin. HeLa cells were plated on the coverslips in complete growth medium and allowed to adhere for 12 hours. Transfection of HeLa cells was performed with Lipofectamine 2000 (Invitrogen, Carlsbad, CA). Cells were transfected with 0.8 μg of DNA and 2 μl Lipofectamine 2000 in normal growth medium for 24 hours. Cells were then fixed with 4% paraformadehyde, permeabilized briefly on ice with 1% Triton X-100, and labeled with either anti-vinculin antibody at a dilution of 1:300 (V9131, Sigma) or FITC-phalloidin at 1:400 dilution as previously described in our analysis of the subcellular targeting of mammalian Talin1 [13]. Fluorescence images were obtained with a Zeiss Axiovert 200 microscope and processed using OpenLab (Improvision). Expression of intact dsRed-Talin-a/b constructs in transfected cells was confirmed in immunoblots using an anti-dsRed polyclonal antibody. We have shown previously that the addition of a fluorescence tag to the N-terminus of both full-length talin and talin I/LWEQ module constructs has no effect on the talin subcellular targeting [10,13]. Transfection of HeLa cells with the dsRed vector alone did not affect the morphology of HeLa cells and dsRed protein was diffusely distributed throughout the cytoplasm.

## Authors' contributions

RM conceived of this work, provided advice on the design of the experiments, and provided funding. RS executed the experiments in this study. The manuscript was written by both authors.

## Supplementary Material

Additional File 1 Alignment of full-length chordate talin protein sequences. **A. **Chordate talin sequence alignment. Talin1 and Talin2 from human (Hs, *Homo sapiens*), chicken (Gg, *Gallus gallus*), pufferfish (Tn, *Tetraodon nigroviridis*), and *Ciona intestinalis *(Ci Tn-a/b) were aligned using CLUSTAL W. The *T. nigroviridis *sequences are from the Genoscope database [39] and have been annotated manually. The *G. gallus *Talin2 sequence was compiled manually from the current version of the chicken genome (GenBank). The gap at position 1789–1830 corresponds to exon 40 of the human Talin1/2 sequences and may represent an unsequenced region of the chicken genome, or chicken Talin2 may lack this exon altogether. The insertion at position 12–13 shows that *C. intestinalis *talin is orthologous to vertebrate Talin2. The I/LWEQ module begins at position 2345 of human Talin1 (ILEAAK). The alternative splice variants of *C. intestinalis *Talin-a and Talin-b are shown in red. Sequence identity (*); sequence similarity (:). An unrooted tree based on this alignment is shown in Fig. 1. **B. **Identity/similarity matrix of chordate talins. Percent sequence identity is shown below the diagonal; percent sequence similarity is shown above the diagonal. Matrix values were calculated using MacBoxShade. Click here for file

Additional File 2 Alignment of *C. intestinalis *Talin-a protein sequences. Protein sequences are from the EST sequences listed in Table 1. The alternatively spliced exon sequence is in red. Click here for file

Additional File 3 Alignment of *C. intestinalis *Talin-b protein sequences. Protein sequences are from the EST sequences listed in Table 1. The alternatively spliced exon sequence is in red. Click here for file
