# Supplementary material for: Evidence that talin alternative splice variants from Ciona intestinalis have different roles in cell adhesion
Source: BMC Cell Biol. 2006 Dec 6;7:40. doi: 10.1186/1471-2121-7-40 (PMC1702346; doi:10.1186/1471-2121-7-40)
Supplement: Additional File 1 — Alignment of full-length chordate talin protein sequences. A. Chordate talin sequence alignment. Talin1 and Talin2 from human (Hs, Homo sapiens), chicken (Gg, Gallus gallus), pufferfish (Tn, Tetraodon nigroviridis), and Ciona intestinalis (Ci Tn-a/b) were aligned using CLUSTAL W. The T. nigroviridis sequences are from the Genoscope database [39] and have been annotated manually. The G. gallus Talin2 sequence was compiled manually from the current version of the chicken genome (GenBank). The gap at position 1789–1830 corresponds to exon 40 of the human Talin1/2 sequences and may represent an unsequenced region of the chicken genome, or chicken Talin2 may lack this exon altogether. The insertion at position 12–13 shows that C. intestinalis talin is orthologous to vertebrate Talin2. The I/LWEQ module begins at position 2345 of human Talin1 (ILEAAK). The alternative splice variants of C. intestinalis Talin-a and Talin-b are shown in red. Sequence identity (*); sequence similarity (:). An unrooted tree based on this alignment is shown in Fig. 1. B. Identity/similarity matrix of chordate talins. Percent sequence identity is shown below the diagonal; percent sequence similarity is shown above the diagonal. Matrix values were calculated using MacBoxShade. [file 1471-2121-7-40-S1.doc]

**Additional File 1**

**A.**

Hs Talin1 1 MVALSLKISIG--NVVKTMQFEPSTMVYDACRIIRERIPEAPAGPPSDFGLFLSDDDPKKGIWLEAGKALDYYMLRNGDTMEYRKKQRPLKIRMLDGTVK 98

Gg Talin1 1 MVALSLKISIG--NVVKTMQFEPSTMVYDACRMIRERVPEAQMGQPNDFGLFLSDEDPKKGIWLEAGKALDYYMLRNGDTMEYKKKQRPLKIRMLDGTVK 98

Tn Talin1 1 MVALSLKIGVG--NVVKAMQFEPSTMVYDACRIIRERVPEAQMGQPNDYGLFLSDEDPKKGIWLEAGKALDYYMLRNGDTLEYKKKQRPLKIRMLDGTVK 98

Hs Talin2 1 MVALSLKICVRHCNVVKTMQFEPSTAVYDACRVIRERVPEAQAGQASDYGLFLSDEDPRKGIWLEAGRTLDYYMLRNGDILEYKKKQRPQKIRMLDGSVK 100

Gg Talin2 1 MVALSLKICVRQCNVVKTMQFEPSTAVYDACRVIRERVPEAQMGQASDYGLFLSDEDPRKGIWLEAGRTLDYYMLRNGDVLEYKKKQRPQKIRMLDGSVK 100

Tn Talin2 1 MVVLSLKICVRQCNVVKTMQFEPSTAVYDACRIIRERVPEAQTGQASDHGLFLSDEDPRKGIWLESGRTLDYYMLRNGDILEYKKKQRPQKIKMLDGAIK 100

Ci Talin-a 1 MVHLNLKIHIKQSSMTRTMQFDPACIVYDACRIIREKSPEAQVGQAQDYSLFLADKDPKKGVWLESRRPLEYYLLRDGDILEYKQKQRPLKVRTLDASIK 100

Ci Talin-b 1 MVHLNLKIHIKQSSMTRTMQFDPACIVYDACRIIREKSPEAQVGQAQDYSLFLADKDPKKGVWLESRRPLEYYLLRDGDILEYKQKQRPLKVRTLDASIK 100

** * *** : : ::***:*:: ******:***: *** * * ***:* **:**:***: : *:**:** ** :**::**** *:: ** ::*

Hs Tn1 99 TIMVDDSKTVTDMLMTICARIGITNHDEYSLVRELMEEKKEEITGTLRKDKTLLRDEKKMEKLKQKLHTDDELNWLDHGRTLREQGVEEHETLLLRRKFF 198

Gg Tn1 99 TVMVDDSKTVTDMLTTICARIGITNYDEYSLVREIMEEKKEEVTGTLKKDKTLLRDEKKMEKLKQKLHTDDELNWLDHGRTLREQGIDDNETLLLRRKFF 198

Tn Tn1 99 TVMVDDSKIVSDMLMTICARIGITNYDEYSLVRDMGEEKKEETTGTLRKDKTLLRDDKKMEKLKQKLHTDDELNWLDHGRTLREQGVEETEMLLLRRKFF 198

Hs Tn2 101 TVMVDDSKTVGELLVTICSRIGITNYEEYSLIQETIEEKKEEGTGTLKKDRTLLRDERKMEKLKAKLHTDDDLNWLDHSRTFREQGVDENETLLLRRKFF 200

Gg Tn2 101 TVMVDDSKTVGELLVTICSRIGITNYEEYSLIQESIEEKKEESTGTLKKDRTLLRDERKMEKLKAKLHTDDDLNWLDHSRTFREQGVDENETLLLRRKFF 200

Tn Tn2 101 TIMLDDSKTVGELLVTICSRIGITNYEEYSLIQETVEEKKEDGMGTLKKDRTLLRDERKMEKLKAKLHTDDDLNWLDHSRTFREQGVDESETLLLRRKFF 200

Ci Tn-a 101 TVMIDDSNTVDQLMITICTRLGIVNHEEYSLVRDTIVDEAPLQKKDTGTLLRPGTTDRKFETLKKKLHTDDELNWLSHGQTLREQGVEEFETLVLRRKYF 200

Ci Tn-b 101 TVMIDDSNTVDQLMITICTRLGIVNHEEYSLVRDTIVDEAPLQKKDTGTLLRPGTTDRKFETLKKKLHTDDELNWLSHGQTLREQGVEEFETLVLRRKYF 200

*:*:*** * :: ***:*:** * :****::: : :: * * ** ******:**** * :* ****::: * *:****:*

Hs Tn1 199 YSDQNVDSRDPVQLNLLYVQARDDILNGSHPVSFDKACEFAGFQCQIQFGPHNEQKHKAGFLDLKDFLPKEYVKQK-GERKIFQAHKNCGQMSEIEAKVR 297

Gg Tn1 199 YSDQNVDSRDPVQLNLLYVQARDDILNGSHPVSFDKACEFAGYQCQIQFGPHNEQKHKPGFLELKDFLPKEYIKQK-GERKIFMAHKNCGNMSEIEAKVR 297

Tn Tn1 199 YSDQNVDSRDPVQLNLLYVQARDDILNGSHPVSFDKACEFAGYQCQIQFGDHNESKHKSGFLDLKEFLPKEYIKNK-GEKRIFQAHKNCQNMTEIEAKVS 297

Hs Tn2 201 YSDQNVDSRDPVQLNLLYVQAQDDILNGSHPVSFEKACEFGGFQAQIQFGPHVEHKHKPGFLDLKEFLPKEYIKQRGAEKRIFQEHKNCGEMSEIEAKVK 300

Gg Tn2 201 YSDQNVDSRDPVQLNLLYVQARDDILNGSHPVSFEKACEFGGFQAQIQFGPHVEHKHKPGFLDLKEFLPKEYTKQRGAEKRIFQEHKNCGEMTEIEAKVK 300

Tn Tn2 201 YSDQNVDSRDPVQLNLLYVQARDDILNGSHPVSFDKACEFGGIQAQIQFGPHIEHKHKPGFLDLKEFLPKEYIKQRGAEKKIFQEHKNCGEMTEIEAKVK 300

Ci Tn-a 201 YSDQNVDSRDPIQLNLLYVQSRDGILKGQYPVSEKDATTFAAIQCQIQLGNHDEKKHKPGYIELKDFLPKEYVKSRGIEKKIFAEHKAFESLNEIEAKVK 300

Ci Tn-b 201 YSDQNVDSRDPIQLNLLYVQSRDGILKGQYPVSEKDATTFAAIQCQIQLGNHDEKKHKPGYIELKDFLPKEYVKSRGIEKKIFAEHKAFESLNEIEAKVK 300

***********:********::* ** * *** *: * *:*** * * * *** *:::**:****** * : *::** ** : ******

Hs Tn1 298 YVKLARSLKTYGVSFFLVKEKMKGKNKLVPRLLGITKECVMRVDEKTKEVIQEWNLTNIKRWAASPKSFT------LDFGDYQDGYYSVQTTEGEQIAQL 391

Gg Tn1 298 YVKLARSLKTYGVSFFLVKEKMKGKNKLVPRLLGITKECVMRVDEKTKEVIQEWSLTNIKRWAASPKSFT------LDFGDYQDGYYSVQTTEGEQIAQL 391

Tn Tn1 298 YVKLARSLKTYGVSFFLVKEKMKGKNKLVPRLLGITKESVMRVDEKTKEVIQEWNLTNIKRWAASPKSFT------LDFGDYQDGYYSVQTTEGEQIAQL 391

Hs Tn2 301 YVKLARSLRTYGVSFFLVKEKMKGKNKLVPRLLGITKDSVMRVDEKTKEVLQEWPLTTVKRWAASPKSFT------LDFGEYQESYYSVQTTEGEQISQL 394

Gg Tn2 301 YVKLARSLRTYGVSFFLVKEKMKGKNKLVPRLLGVTKDSVMRVDEKTKEVLQEWPLTTVKRWAASPKSFT------LDFGEYQESYYSVQTTEGEQISQL 394

Tn Tn2 301 YVKLARSLRTYGVSFFLVKEKMKSKNKLVPRLLGITKESVMRVDERTKDVVQEWPLTTVKRWAASPKSFTPGERSKHDFGEYQESYYSVQTTEGEQISQL 400

Ci Tn-a 301 YTKNCRALKTYGVTFFLVKEKMKGRNKLVPRLMGVTRESVMRVDEKTKDMLKVWPLTSVKRWAASPKSFT------LDFGDYQDGYYSVQTTEGEQIAQL 394

Ci Tn-b 301 YTKNCRALKTYGVTFFLVKEKMKGRNKLVPRLMGVTRESVMRVDEKTKDMLKVWPLTSVKRWAASPKSFT------LDFGDYQDGYYSVQTTEGEQIAQL 394

* * :*:*:****:********* :*******:*:*:::******:**:::: * ** :*********** ***:**: ************:**

Hs Tn1 392 IAGYIDIILKKKKSKDHFGLEGDEESTMLEDSVSPKKSTVLQQQYNRVGKVEHGSVALPAIMRSGASGPENFQVGSMPPAQQQITSGQMHRGHMPPLTSA 491

Gg Tn1 392 IAGYIDIILKKKKSKDHFGLEGDEESTMLEDSVSPKKSTVLQQQFNRVGKAELGSVALPAIMRTGAAGPENFQVGTMPQAQMQITSGQMHRGHMPPLTSA 491

Tn Tn1 392 IAGYIDIILKKKKSKDHFGLEGDEESTMLEDSVSPKKSTVMQQQCNKVGKVETGSVALPAIMRSGAAGPESFQMGSMPQAKQHVTSGQMHRGHMPPLTSA 491

Hs Tn2 395 IAGYIDIILKKKQSKDRFGLEGDEESTMLEESVSPKKSTILQQQFNRTGKAEHGSVALPAVMRSGSSGPETFNVGSMPSPQQQVMVGQMHRGHMPPLTSA 494

Gg Tn2 395 IAGYIDIILKKKQSKDRFGLEGDEESTMLEESVSPKKSTILQQQFNRAGKVEHGSVALPAVMRSGSSGPETFNVGIMPSPQQQVTIGQMHRGHMPPLTSA 494

Tn Tn2 401 IAGYIDIILKKKQSKDRFGLEGDEESTMLEESVSPKKSTILQQQFNRVGRVEHGSVALPGVIRSGSIGTESLSMGTMPCAQQQITMGQMHRGHMPPLSSA 500

Ci Tn-a 395 IAGYIDIILKKRQAKDNYGPDADEDAAMVEDVVSPHRAQLVAMHGGSAGSFHSGSVAMPGVIRNSSARPDSYSMGAMQPPTQVTTHQNLSFGGQP-LTAA 493

Ci Tn-b 395 IAGYIDIILKKRQAKDNYGPDADEDAAMVEDVVSPHRAQLVAMHGGSAGSFHSGSVAMPGVIRNSSARPDSYSMGAMQPPTQVTTHQNLSFGGQP-LTAA 493

***********:::** :* : **:::*:*: ***::: :: : : * ****:* ::* : : :* * :: * * *::*

Hs Tn1 492 QQALTGTINSSMQAVQAAQATLDDFDTLPPLGQDAASKAWRKNKMDESKHEIHSQVDAITAGTASVVNLTAGDPAETDYTAVGCAVTTISSNLTEMSRGV 591

Gg Tn1 492 QQALTGTINSSMQAVNAAQATLDDFETLPPLGQDAASKAWRKNKMDESKHEIHSQADAITAGTASVVNLTAGDPADTDYTAVGCAVTTISSNLTEMSKGV 591

Tn Tn1 492 QQALTGTINSSMQAVQAAQASLDDCETLPPLGTDAASQAWRRNKMDESKHEIHSQVDAITAGTASMVNLTGGDPAETDYTAVGCAITTISSNLTEMSKGV 591

Hs Tn2 495 QQALMGTINTSMHAVQQAQDDLSELDSLPPLGQDMASRVWVQNKVDESKHGIHSQVDAITAGTASVVNLTAGDPADTDYTAVGCAITTISSNPTEMSKGV 594

Gg Tn2 495 QQALMGTINTSMHAVQQAQADLSEVDNLPPLGQDMASRVWVQNKVDESKHEIHSQVDAITAGTASVVNLTAGDPVDTDYTAVGCAITTISSNLTEMSKGV 594

Tn Tn2 501 QQALMGTINTSMQAVQKAQIDLDEVDNLPPLGQDMASKVWIQNKMDESKHEIHSQVDAITAGTASVVNLTAGDPTDTDYTAVGCAITTISSNLTEMSKGV 600

Ci Tn-a 494 QQAFMGNIEHGFNACRAAHEGFNNKANLPPLGSDAASKQWKLNALEENRHNIQSNLAAIDAATATVITKTSGDREGTNYTTVGSAITTISSNLNDMTKSL 593

Ci Tn-b 494 QQAFMGNIEHGFNACRAAHEGFNNKANLPPLGSDAASKQWKLNALEENRHNIQSNLAAIDAATATVITKTSGDREGTNYTTVGSAITTISSNLNDMTKSL 593

*** *:* * *: ***** * **: * * ::* :* *:*: ** * **:::: * ** * **:**:*:****** ::*:: :

Hs Tn1 592 KLLAALLEDEGGSGRPLLQAAKGLAGAVSELLRSAQPASAE--PRQNLLQAAGNVGQASGELLQQIGESDTDPHFQDALMQLAKAVASAAAALVLKAKSV 689

Gg Tn1 592 KLLAALMEDEGGNGRQLLQAAKNLASAVSDLLKTAQPASAE--PRQNLLQAAGLVGQTSGELLQQIGESDTDPRFQDMLMQLAKAVASAAAALVLKAKNV 689

Tn Tn1 592 KLLAALMDDEGGSGQQLLGAAKNLACAVSDMLKTAQPASTEVAPRQNLLQAAGNVGQASGELLSHIGETDTDPQFQDMLMQLAKAVANAAAALVLKAKNV 691

Hs Tn2 595 KLLAALMDDEVGSGEDLLRAARTLAGAVSDLLKAVQPTSGE--PRQTVLTAAGSIGQASGDLLRQIGENETDERFQDVLMSLAKAVANAAAMLVLKAKNV 692

Gg Tn2 595 KLLAALMDDEGGSGEDLLKAARTKGIAVSDLLKAVQPTSGX--PRQTVLTAAGSIGQASGELLRQIGENETDERFQDVLMSLAKAVANAAAMLVLKAKNV 692

Tn Tn2 601 KLLAALMEDDVGGGNDLMRAARTLAGAVSDLLKAVEPASGE--PRQTVLTAAGSIGQASGDLLRQIGENETDERFQDILMNLAKAVANAAAMLVLKAKNV 698

Ci Tn-a 594 RLMAALLDD-GRDGDGLMKAARDLTAAIQDLLKAAQPNSEE--PRQNLLGAAGKIGDASHDILKYIGEG-GDDEFQDILMNLAKKVANATAALVLKGKTV 689

Ci Tn-b 594 RLMAALLDD-GRDGDGLMKAARDLTAAIQDLLKAAQPNSEE--PRQNLLGAAGKIGDASHDILKYIGEG-GDDEFQDILMNLAKKVANATAALVLKGKTV 689

:*:***::* * *: **: *: ::*:: :* * ***::* *** :* :* ::* *** * *** ** *** ** *:* **** * *

Hs Tn1 690 AQRTEDSGLQTQVIAAATQCALSTSQLVACTKVVAPTISSPVCQEQLVEAGRLVAKAVEGCVSASQAATEDGQLLRGVGAAATAVTQALNELLQHVKAHA 789

Gg Tn1 690 AQKTEDSALQTQVIAAATQCALSTSQLVACTKVVAPTISSPVCQEQLIEAGKLVAKSAEGCVEASKAATNDDQLLKQVGVAATAVTQALNDLLQHIKQHA 789

Tn Tn1 692 AQKSEDPAQQNRVIAAATQCALSTSQLVACTRVVAPTISSPVCQEQLIEASKLVAKSVEGCVEASQGATGDEGLLKQVGVAATGVTHALNDLLQHIKQYA 791

Hs Tn2 693 AQVAEDTVLQNRVIAAATQCALSTSQLVACAKVVSPTISSPVCQEQLIEAGKLVDRSVENCVRACQAATTDSELLKQVSAAASVVSQALHDLLQHVRQFA 792

Gg Tn2 693 AQVAEDTVLQNRVIAAATQCALSTSQLVACAKVVSPTISSPVCQEQLIEAGKLVDRSVENCVRACQAATDDTELLKQVSAAASIVSQALNDLLQHVRQFA 792

Tn Tn2 699 AQVAEDTVLQNRVIAAATQCALSTSQLVACAKVVSPTISSPVCQEQLIEAGKLVDRSVESCIQACLSATEDGELLKQVSAAASVVGQALEDLLQHVRQYT 798

Ci Tn-a 690 ASQSNDQQVQNQVIASATQCALSTSQLVACTKVVGPTISNPSCKEQLIDAAREVSQSVEGCLESTQIATGDPDLLRALGDAASNVTQALNDLINHIKMVG 789

Ci Tn-b 690 ASQSNDQQVQNQVIASATQCALSTSQLVACTKVVGPTISNPSCKEQLIDAAREVSQSVEGCLESTQIATGDPDLLRALGDAASNVTQALNDLINHIKMVG 789

* : * *::***:**************::** **** * *:***::* : * :: * *: :: ** * **: : **: * :** :*::*::

Hs Tn1 790 TGAGPAGRYDQATDTILTVTENIFSSMGDAGEMVGQARILAQATSDLVNAIKADAEGESDLENSRKLLSAAKILADATAKMVEAAKGAAAHPDSEEQQQR 889

Gg Tn1 790 TGGQPIGRYDQATDTILNVTENIFSSMGDAGEMVRQARILAQATSDLVNAIKADAEGETDLENSRKLLSAAKILADATAKMVEAAKGAAAHPDSEEQQQR 889

Tn Tn1 792 SGATAMGRHGEATDRILDVTENIFSSMGDAGEMVRQARILAQATSDLVNAIKMDAEGESDLENSRKLLSAAKLLADATAKMVEAAKGAAANPDSEEQQQK 891

Hs Tn2 793 SRGEPIGRYDQATDTIMCVTESIFSSMGDAGEMVRQARVLAQATSDLVNAMRSDAEAEIDMENSKKLLAAAKLLADSTARMVEAAEGAAANPENEDQQQR 892

Gg Tn2 793 SRGEPIGRYDQATDTIMCVTESIFSSMGDAGEMVRQARVLAQATSDLVNAMRSDAEAEIDMENSKKLLAAAKLLADSTARMVEAAKGAAANPENEDQQQR 892

Tn Tn2 799 ARGEPIGRYDQATDTIMTVTESIFSSMGDAGEMVRQARVLAQATSDLVNAMRSDAEAEVDVDNSKKLLAAAKLLADATARMVEAAKGAAAYPENEDQQQR 898

Ci Tn-a 790 D-----GKYDDQCEAILNATDKLCNSMGNAAVMAKQARLLGQTTSDLVNLLRLEAEDAQDDDWRKWLLSTVKLVADATAKMVEAAKGTASNPHDSQQQQK 884

Ci Tn-b 790 D-----GKYDDQCEAILNATDKLCNSMGNAAVMAKQARLLGQTTSDLVNLLRLEAEDAQDDDWRKWLLSTVKLVADATAKMVEAAKGTASNPHDSQQQQK 884

*: :: *: *: : *** * * ***:* *:****** :: :** * : : **:: *::**:**:***** *:*: * ***:

Hs Tn1 890 LREAAEGLRMATNAAAQNAIKKKLVQRLEHAAKQAAASATQTIAAAQHAASTPKASAGPQPLLVQSCKAVAEQIPLLVQGVRGSQAQPDSPSAQLALIAA 989

Gg Tn1 890 LREAAEGLRMATNAAAQNAIKKKLVHKLEHAAKQAAASATQTIAAAQHAAASNKN-PAAQQQLVQSCKVVADQIPMLVQGVRGSQSQPDSPSAQLALIAA 988

Tn Tn1 892 LREAAEGLRMATNAAAQNAIKKRLVSKLESAAKHAAAAATQTIAAAQHAASSNKN-QAAQQQLVQSCKVVAEQIPQLVQGVRGSQAQPDSPSAQLALIRA 990

Hs Tn2 893 LREAAEGLRVATNAAAQNAIKKKIVNRLEVAAKQAAAAATQTIAASQNAAVSNKN-PAAQQQLVQSCKAVADHIPQLVQGVRGSQAQAEDLSAQLALIIS 991

Gg Tn2 893 LREAAEGLRVATNAAAQNAIKKKIVNRLEIAAKQAAAAATQTIAASQNAAVSNKN-TAAHQQLVQSCKNVADHIPQLVQGVRGSQAQAEDLSAQLALINS 991

Tn Tn2 899 LREAAEGLRVATNAAAQNAIKKKLINRLENAAKQAAAAATQTIAAAQNAAASNKN-TAAHQQLVQSCKAVADHIPQLVQGVRSSQASPEDLSAQLALIIA 997

Ci Tn-a 885 LKLAAENLRAATNAATQNALRKKLVRRLEQAASQAAVAATQTIAAANAAEPHNTNKTSQNQLLTHSKTLQSDHIPKLLQGIHGAYEDLENPTVQQNLITA 984

Ci Tn-b 885 LKLAAENLRAATNAATQNALRKKLVRRLEQAASQAAVAATQTIAAANAAEPHNTNKTSQNQLLTHSKTLQSDHIPKLLQGIHGAYEDLENPTVQQNLITA 984

*: ***:** *****:***::*::: :** ** :** :*******:: * * :* :::** *:**:: : : : * ** :

Hs Tn1 990 SQSFLQPGGKMVAAAKASVPTIQDQASAMQLSQCAKNLGTALAELRTAAQKAQEACGPLEMDSALSVVQNLEKDLQEVKAAARDGKLKPLPGETMEKCTQ 1089

Gg Tn1 989 SQNFLQPGGKMVAAAKATVPTITDQASAMQLSQCAKNLAAALAELRTAAQKAQEACGPLEIDSALGLVQSLERDLKEAKAAARDGKLKPLPGETMEKCAQ 1088

Tn Tn1 991 SQNFLQPGAKMVAASKATVPTITDQASAMQLSQCAKNLAGALAELRTASQKAQEACGPLEIDNALSTVRKLEKDIQESKASAKEGRLRPLPGETLDKCSQ 1090

Hs Tn2 992 SQNFLQPGSKMVSSAKAAVPTVSDQAAAMQLSQCAKNLATSLAELRTASQKAHEACGPMEIDSALNTVQTLKNELQDAKMAAVESQLKPLPGETLEKCAQ 1091

Gg Tn2 992 SQNFLQPGSKMVASAKAAVPTVTDQAAAMQLSQCAKNLATSLAELRTASQKAHEACGPMEIDSALNTVQTLKSELQDAKMAAVDGQLKPLPGETLEKCAQ 1091

Tn Tn2 998 SQNFLQPGSKMVTSAKSSVPTVTDQAAAMQLGQCAKNLATCLAELRTSAQKAHEACGPMEIDSALTAIQTLRSELQDAKMAAVNTQLKPLPGESLEKCAQ 1097

Ci Tn-a 985 SNEFIPPATKMVAYSKAVVPTVSEKSTALQLANCTKKLALAVAELKTAAVKAGEVCGASGIDAALETVTALDRQLSVYCADAKNGELQPLPGQTMQSCAQ 1084

Ci Tn-b 985 SNEFIPPATKMVAYSKAVVPTVSEKSTALQLANCTKKLALAVAELKTAAVKAGEVCGASGIDAALETVTALDRQLSVYCADAKNGELQPLPGQTMQSCAQ 1084

*: *: * ***: :*: ***: ::::*:** :*:* * ::***:*:: ** * ** :* ** : * : * *:****::: *:*

Hs Tn1 1090 DLGNSTKAVSSAIAQLLGEVAQGNENYAGIAARDVAGGLRSLAQAARGVAALTSDPAVQAIVLDTASDVLDKASSLIEEAKKAAGHPGDPESQQRLAQVA 1189

Gg Tn1 1089 DLGNSTKAVTSAIAHLLGEVAQGNENYTGIAAREVAQALRSLSQAARGVAANSSDPQAQNAMLECASDVMDKANNLIEEARKAVAKPGDPDSQQRLVQVA 1188

Tn Tn1 1091 DLGNSTKAVSSAMAQLLSEATQGNENYTGMAARDVAQALKTFASASRGVAATTEEPSARNAVLDCAADVLDKSANLIEETKRAVVKPGDAEGQQRLAQVA 1190

Hs Tn2 1092 DLGSTSKAVGSSMAQLLTCAAQGNEHYTGVAARETAQALKTLAQAARGVAASTTDPAAAHAMLDSARDVMEGSAMLIQEAKQALIAPGDAERQQRLAQVA 1191

Gg Tn2 1092 DLGSTSKAVGSSMAQLLTCAAQGNEHYTGVAARETAQALKTLAQAARGVAASTTDPVAAHAMLDSARDVMEGSAMLIQEAKQALAAPGDADSQQRLAQVA 1191

Tn Tn2 1098 DLGSTSKSVGSSMAQLLTCAAQGNEHYTGIAARETAQALKTLAQAARGVAASTTDPKAAAAMLDSARDVMEGSALLIEEAKQALVSPGDAESQQRLAQVA 1197

Ci Tn-a 1085 ELGATSKAVGSSMAQLLTAAAQGNEDYTGMAARNTANALRTLVGAARGVSANLPDLESQLNLLETCRDVMDKSVNLMQEAKLAVEDPENPENRQRLAQVA 1184

Ci Tn-b 1085 ELGATSKAVGSSMAQLLTAAAQGNEDYTGMAARNTANALRTLVGAARGVSANLPDLESQLNLLETCRDVMDKSVNLMQEAKLAVEDPENPENRQRLAQVA 1184

:** ::*:* *::*:** :**** *:*:*** * *:: *:***:* : :*::: **:: : *::*:: * * : :*** ***

Hs Tn1 1190 KAVTQALNRCVSCLPGQRDVDNALRAVGDASKRLLSDSLPPSTGTFQEAQSRLNEAAAGLNQAATELVQASRGTPQDLARASGRFGQDFSTFLEAGVEMA 1289

Gg Tn1 1189 KAVSQALNRCVNCLPGQRDVDAAIRMVGEASKRLLSDSFPPSNKTFQEAQSQLNRAAAGLNQSANELVQASRGTPQDLAKSSGKFGQDFNEFLQAGVEMA 1288

Tn Tn1 1191 KAVSQALNRCVNCLPGQRDVDNAIRSVGEASKTLLNESFPSSGRSFQEVQAQLNEVAVCLNQSANEVVQASRGTTLDLAKATSKFGKDFGSFLEAGVDMA 1290

Hs Tn2 1192 KAVSHSLNNCVNCLPGQKDVDVALKSIGESSKKLLVDSLPPSTKPFQEAQSELNQAAADLNQSAGEVVHATRGQSGELAAASGKFSDDFDEFLDAGIEMA 1291

Gg Tn2 1192 KAVSHSLNNCVNCLPGQKDVDVALKSIGESSKKLLVDLLPPSSKSFQEAQSELNQAAADLNQSAGEVVHATRGQSGELAAASGKFSDDFDEFLDAGIEMA 1291

Tn Tn2 1198 KAVSHSLNNCVNCLPGQKDVDMALKSIGEASKKLLIETIPPASKSFQEAQNELNHTAEELNQSAGEVVHASRGPSSQLAVASGKFSEDFDEFLDAGLEMA 1297

Ci Tn-a 1185 KAVSHALNNCINCLPGQRDVDEALKNIAESSKRLLSNQFPVTNSNFQTAQAQLNKTAEELNIAANDLVGASRGTPSELAASSCNYNDRFTELLDAGMNVA 1284

Ci Tn-b 1185 KAVSHALNNCINCLPGQRDVDEALKNIAESSKRLLSNQFPVTNSNFQTAQAQLNKTAEELNIAANDLVGASRGTPSELAASSCNYNDRFTELLDAGMNVA 1284

***:::** *: *****:*** *:: : ::** ** * : ** * ** * ** :* ::* *:** ** :: : * * **: :*

Hs Tn1 1290 GQAPSQEDRAQVVSNLKGISMSSSKLLLAAKALSTDPAAPNLKSQLAAAARAVTDSINQLITMCTQQAPGQKECDNALRELETVRELLENPVQPINDMSY 1389

Gg Tn1 1289 SLSPTKEDQAQVVSNLKSISMSSSKLLLAAKALSADPTSPNLKSQLAAAARAVTDSINQLITMCTQQAPGQKECDNALRELETVKELLENPTQTVNDMSY 1388

Tn Tn1 1291 GTSPSKEDQGQVVTNLKTISMSSSKLLLAAKALSTDPGSPNLKNQLAAAARAVTDSINQLITMCTQQAPGQKECDNALRELESVVGMLENPTQAVSDASY 1390

Hs Tn2 1292 GQAQTKEDQIQVIGNLKNISMASSKLLLAAKSLSVDPGAPNAKNLLAAAARAVTESINQLITLCTQQAPGQKECDNALRELETVKGMLDNPNEPVSDLSY 1391

Gg Tn2 1292 GQAQTKEDQIQVIGNLKSISMASSKLLLAAKSLSVDPGAPNAKNLLAAAARAVTESINQLITLCTQQAPGQKECDNALRELETVKGMLDNPNEPVSDLSY 1391

Tn Tn2 1298 GHTQKKDDQVQVIGSLKNISMASSKLLLAAKSLSVDPAAANAKNLLAAAARAVTDSINQLITLCTQQAPGQKECDNALRELEAVRGMLDHPNEPVSDLSY 1397

Ci Tn-a 1285 GQSRDKEDQNHVVGNLKSISMASSKLLLAAKALSADPGAPNAKNQLSAAARAVTESINNLITHCTETAPGQKECDNALRQLKTVKEMLENPNEPVNDFSY 1384

Ci Tn-b 1285 GQSRDKEDQNHVVGNLKSISMASSKLLLAAKALSADPGAPNAKNQLSAAARAVTESINNLITHCTETAPGQKECDNALRQLKTVKEMLENPNEPVNDFSY 1384

: ::*: :*: ** ***:*********:** ** : * * *:*******:***:*** **: ************:* :* :*::* : : * **

Hs Tn1 1390 FGCLDSVMENSKVLGEAMTGISQNAKNGNLPEFGDAISTASKALCGFTEAAAQAAYLVGVSDPNSQAGQQGLVEPTQFARANQAIQMACQSLGEPGCTQA 1489

Gg Tn1 1389 FSCLDSVMENSKVLGESMAGISQNAKNSKLPEFGESISAASKALCGLTEAAAQAAYLVGVSDPNSQAGQQGLVDPTQFARANQAIQMACQNLVDPACTQS 1488

Tn Tn1 1391 FDCIDAVMENSKVLGESMAGISHNAKNSNLPEFGDSISSGSKALCGLTEAAAQAAYLVGVSDPNSSAGQKGLVDPAQFARANQSIQMACQNLVDPACTQS 1490

Hs Tn2 1392 FDCIESVMENSKVLGESMAGIPQNAKTGDLPAFGECVGIASKALCGLTEAAAQAAYLVGISDPNSQAGHQGLVDPIQFARANQAIQMACQNLVDPGSSPS 1491

Gg Tn2 1392 FDCIEGVMENSKALGESMAGISQNAKTGDLLVFGECVGVASKALCGLTEAAAQAAYLVGISDPNSQAGQQGLVDPIQFARANQAIQMACQNLVDPASSPS 1491

Tn Tn2 1398 FDCIESVMENSKVLGESMAGISMNCKNGDVAAFGDCVGSASRALCGLTEAAGQAAYLVGVSDPNSQAGHQGLVDPVQFAKANQAIQMACQNLVDPESSPS 1497

Ci Tn-a 1385 FDCLESVMDNSKMLGESMSGITQHARASELESFGEAVTATQKSLIGLTEAAAQAAYLVGIADPNSEAGTQGLVDQTQFARANQAIQMACQSLLDPSSNQP 1484

Ci Tn-b 1385 FDCLESVMDNSKMLGESMSGITQHARASELESFGEAVTATQKSLIGLTEAAAQAAYLVGIADPNSEAGTQGLVDQTQFARANQAIQMACQSLLDPSSNQP 1484

* *:: **:*** ***:*:** ::: : **::: ::* * **** *******::**** ** :***: ***:***:****** * :* :

Hs Tn1 1490 QVLSAATIVAKHTSALCNSCRLASARTTNPTAKRQFVQSAKEVANSTANLVKTIKALDGPFTEENRAQCRAATAPLLEAVDNLSAFASNPEFSSIPAQIS 1589

Gg Tn1 1489 QVLSAATIVAKHTSALCNTCRLASSRTANPVAKRQFVQPAKEVANSTANLVKTIKALDGAFNEENRERCRAATAPLIEAVDNLTAFASNPEFATVPAQIS 1588

Tn Tn1 1491 QVLSAATIVAKHTSALCNACRLASSRTSNPVAKRQFVQSAKEVANTTANLVKSIKALDGAFNQENRDKCRAATGPLIEAVDNLTAFASNPEFASIPAHIS 1590

Hs Tn2 1492 QVLSAATIVAKHTSALCNACRIASSKTANPVAKRHFVQSAKEVANSTANLVKTIKALDGDFSEDNRNKCRIATAPLIEAVENLTAFASNPEFVSIPAQIS 1591

Gg Tn2 1492 QVLSAATIVAKHTSALCNACRIASSKTANPVAKRHFVQSAKEVANSTANLVKTIKALDGDFSEENRNKCRIATAPLIEAVENLTAFASNPEFVSIPAQIS 1591

Tn Tn2 1498 QVLSAATIVAKHTSALCNACRLASSKTTNPAAKRHFVQSAKEVANSTANLVKTIKALDGDFSDENRNSCRVATAPLIEAVENLAIFASNPEFASVPAQIS 1597

Ci Tn-a 1485 QVLSAATIVAKYTSALCNVCRVASNKTTNAVARKHFVQSAKEVAHATANLVRTIKALDGNFSEENRANCSQATKPLIDAVESLTTFASNPEFASVPAKIS 1584

Ci Tn-b 1485 QVLSAATIVAKYTSALCNVCRVASNKTTNAVARKHFVQSAKEVAHATANLVRTIKALDGNFSEENRANCSQATKPLIDAVESLTTFASNPEFASVPAKIS 1584

*********** ****** **:** :*:* *:::*** *****::*****::****** * :** * ** **::**: *: ******* ::**:**

Hs Tn1 1590 PEGRAAMEPIVISAQTMLESAGGLIQTARALAVNPRDPPSWSVLAGHSRTVSDSIKKLITSMRDKAPGQLECETAIAALNSCLRDLDQASLAAVSQQLAP 1689

Gg Tn1 1589 PEGRRAMEPIVTSAKTMLESSAGLIQTARSLAVNPKDPPQWSVLAGHSRTVSDSIKKLITNMRDKAPGQRECDEAIDVLNRCMREVDQASLAAISQQLAP 1688

Tn Tn1 1591 PEGYAAMEPILAAAKTMLESSTGLIQTARSLAVNPKDPPRWSVLAGHSRTVSDSIKKLITSMRDKAPGQRECEDAIEVLNSCIRELDQASLAAISQQLTP 1690

Hs Tn2 1592 SEGSQAQEPILVSAKTMLESSSYLIRTARSLAINPKDPPTWSVLAGHSHTVSDSIKSLITSIRDKAPGQRECDYSIDGINRCIRDIEQASLAAVSQSLAT 1691

Gg Tn2 1592 TEGSRAQEPILVSAKTMLESSSLLIKTARSLAINPKDPPTWSVLAGHSHTVSDSIKSLITSIRDKAPGQRECDYSIDGINRCIRDIEQASLAAVSQSLAT 1691

Tn Tn2 1598 RE-----EPILQSACSMLDSSTHLLKTARSLVINPKDPPTWSVLAGHSRTVSDSIKGLITAIRDKAPGQRECDSSIDNINKCIRDIEQASLAAVSQNLPS 1692

Ci Tn-a 1585 DEAREAQRPIIESGKQMLQSSCDLIKTARKLANNPKDPPTWQVMAGHSRVVSDSIKKLIANIRDNAPGQKECDEAIDRINESIQQFNDAALSAMDQSLPA 1684

Ci Tn-b 1585 DEAREAQRPIIESGKQMLQSSCDLIKTARKLANNPKDPPTWQVMAGHSRVVSDSIKKLIANIRDNAPGQKECDEAIDRINESIQQFNDAALSAMDQSLPA 1684

* **: : ** *: *::*** * **:*** * *:****: ****** **: :** **** **: :* :* ::: *:*:*: * *

Hs Tn1 1690 REG---ISQEALHTEMLTAVQEISHLIEPLAHAARAEASQLGHKVSQMAQYFEPLTLAAVGAASKTLSHPQQMALLDQTKTLAESALQLLYTAKEAGGNP 1786

Gg Tn1 1689 REG---ISQEALHNQMITAVQEINNLIEPVASAARAEASQLGHKVSQMAQYFEPLILAAIGAASKTPNHQQQMNLLDQTKTLAESALQMLYTAKEAGGNP 1785

Tn Tn1 1691 RDD---ISMETLHEQMAASVHEISNLIDPVAVAAHSDASQLGHKVSQMASYFEPLVMAAIGTVSKILSSQQQMAVLDQTKTLSESALQMLYTAKEAGGNP 1787

Hs Tn2 1692 RDD---ISVEALQEQLTSVVQEIGHLIDPIATAARGEAAQLGHKVTQLASYFEPLILAAVGVASKILDHQQQMTVLDQTKTLAESALQMLYAAKEGGGNP 1788

Gg Tn2 1692 RDD---ISVEALQEQLTSVVQEIGHLIDPIATAARGEAAQLGHKVTQLASYFEPLVLAAVGVASKTLDHQQQMTVLDQSKTLAESALQMLYAAKEGGGNP 1788

Tn Tn2 1693 RDD---ISLEALQEQLTSTVQEIGHLIDPVSTAARGEASQLGHKVTQLAGYFEPLIKASVGVASKLKDHQQQMTFLDQTKTMAESALQMLYAAKEGGGNP 1789

Ci Tn-a 1685 RDDNSLNGFQAMVSWVVDTVNQISQCVDPLSNAAKQDAAQLGRQVAQMASYFEPLAHATIGAAANSVNHQRQMDILDYSKTLAESALQLMFAAKEGGGNP 1784

Ci Tn-b 1685 RDDNSLNGFQAMVSWVVDTVNQISQCVDPLSNAAKQDAAQLGRQVAQMASYFEPLAHATIGAAANSVNHQRQMDILDYSKTLAESALQLMFAAKEGGGNP 1784

*: ::: : * :* ::*:: **: :*:***::*:*:* ***** *::* : :** ** :**::*****::::*** ****

Hs Tn1 1787 KQAAHTQEALEEAVQMMTEAVEDLTTTLNEAASAAGVVGGMVDSITQAINQLDEGPMGEPEGSFVDYQTTMVRTAKAIAVTVQEMVTKSNTSPEELGPLA 1886

Gg Tn1 1786 KQAAHTQEALEEAVQMMKEAVEDLTTTLNEAASAAGVVGGMVDSITQAINQLDEGPMGEPEGTFVDYQTTMVKTAKAIAVTVQEMVTKSTTNPDELGILA 1885

Tn Tn1 1788 K-AAHMQEALEESVQMMKEAVDDLGATLAEAASAAGAVGGLVDSINHAINKMEETPTLEPEGTFVDYQTTMVKTAKAIAVTVQEMVTKSNTNPDDLGGLA 1886

Hs Tn2 1789 K-AQHTHDAITEAAQLMKEAVDDIMVTLNEAASEVGLVGGMVDAIAEAMSKLDEGTPPEPKGTFVDYQTTVVKYSKAIAVTAQEMMTKSVTNPEELGGLA 1887

Gg Tn2 1789 --------------------------------------------------KLDESTPADPKGTFVDYQTTVVKYSKAIAITAQEMMTKSVTNPEELGGLA 1838

Tn Tn2 1790 K-AFHTHDAIAEAAMLMREAVDDIMVTLNEAASEVG--------------MLDEGTPSAPEGSFVDYQTSMVKHSKAIAVTAQEMITKSVTCPDELGALA 1874

Ci Tn-a 1785 K-ADNTHEAIDEASASMKEAIEDLLKSVQEAPEALVS--GMVDTISSATAILHSNVQANEDDTFADYQTSIMKHCRAIVVTSSDMSMAMNNRPDELATLA 1881

Ci Tn-b 1785 K-ADNTHEAIDEASASMKEAIEDLLKSVQEAPEALVS--GMVDTISSATAILHSNVQANEDDTFADYQTSIMKHCRAIVVTSSDMSMAMNNRPDELATLA 1881

: :* ****:::: ::** :* :* : *::* **

Hs Tn1 1887 NQLTSDYGRLASEAKPAAVAAENEEIGSHIKHRVQELGHGCAALVTKAGALQCSPSDAYTKKELIECARRVSEKVSHVLAALQAGNRGTQACITAASAVS 1986

Gg Tn1 1886 NQLTNDYGQLAQQAKPAALTAENEEIGSHIKRRVQELGHGCAALVTKAGALQCSPSDAYTKKELIESARKVSEKVSHVLAALQAGNRGTQACITAASAVS 1985

Tn Tn1 1887 NQLTNEFGELAGEAKSAALTAENDEIGSHIKKQVGELGSSCTGLVSKAGALQCSPNDPITKKELIDSARKVSEKVSHVLAALQAGNRGTQACITAASAVS 1986

Hs Tn2 1888 SQMTSDYGHLAFQGQMAAATAEPEEIGFQIRTRVQDLGHGCIFLVQKAGALQVCPTDSYTKRELIECARAVTEKVSLVLSALQAGNKGTQACITAATAVS 1987

Gg Tn2 1839 SQMTNDYGHLALQGRMAAATAEPEEIGFQIKTRVQELGHGCIFLVQKAGALQICPTDSYTKRELIECARAVTEKVSLVLSALQAGNKGTQACITAASAVS 1938

Tn Tn2 1875 SQVTGDYSQLAVQGRLAAHTAEPEEIGFQIKTRVQDLGHGCIFLVQKAGALQITPSDSFTKRELIDCARVVTEKVSLVLSALQAGNKGTQACITAANAVS 1974

Ci Tn-a 1882 KKVTEEYSALASEGAAAASLADSNEVSNHIKKSIQDLGDACKELVTASGMVQSNPRDIHAKKDLKDAGKNINEKVSYVMSSLQEGGRGTQACINAHTKVQ 1981

Ci Tn-b 1882 KKVTEEYSALASEGAAAASLADSNEVSNHIKKSIQDLGDACKELVTASGMVQSNPRDIHAKKDLKDAGKNINEKVSYVMSSLQEGGRGTQACINAHTKVQ 1981

::* :: ** : ** *: *: :*: : :** * ** :* :* * * :*::* :: : : **** *:::** *::******:* *

Hs Tn1 1987 GIIADLDTTIMFATAGTLNRE-GTETFADHREGILKTAKVLVEDTKVLVQNAAGSQEKLAQAAQSSVATITRLADVVKLGAASLGAEDPETQVVLINAVK 2085

Gg Tn1 1986 GIIADLDTTIMFATAGTLNRE-NSETFADHREGILKTAKALVEDTKVLVQNATASQEKLAQAAQSSVSTITRLAEVVKLGAASLGSEDPETQVVLINAVK 2084

Tn Tn1 1987 GIIADLDTTIMFATAGTLNRE-NAETFADHREYILKTAKALVEDTKMLVSGAGASQEKLAQAAQSSVSTITKLADVVKLGAASLGSEDPETQVVLINAVK 2085

Hs Tn2 1988 GIIADLDTTIMFATAGTLNAE-NSETFADHRENILKTAKALVEDTKLLVSGAASTPDKLAQAAQSSAATITQLAEVVKLGAASLGSDDPETQVVLINAIK 2086

Gg Tn2 1939 GIIADLDTTIMFATAGTLNAE-NNESFADHRENILKTAKALVEDTKLLVSGAASSQDKLAQAAQSSANTITQLAEVVKLGAASLGSDDPETQVVLINAIK 2037

Tn Tn2 1975 GIIADLDTTIMFASAGTLNAE-NEESFADHRENILKTAKALVEDTKMLVSGAASGQDKLSQAAQSSAKTITQLTDVVKLGATSIGSDDPETQVVLINAVK 2073

Ci Tn-a 1982 GIIGDLDTTLMFVTSGALNPESDNESFAEHRENILSTAKALVEDTKQLVAGAAGGQEKLAGAAQSASQTISKLADVVKSGASSLGADDPDTQVILINAVR 2081

Ci Tn-b 1982 GIIGDLDTTLMFVTSGALNPESDNESFAEHRENILSTAKALVEDTKQLVAGAAGGQEKLAGAAQSASQTISKLADVVKSGASSLGADDPDTQVILINAVR 2081

*** *****:** ::*:** * *:**:*** ** *** ****** ** :* :**: ****: **::*::*** **:*:*::**:***:****::

Hs Tn1 2086 DVAKALGDLISATKAAAGKVGDDPAVWQLKNSAKVMVTNVTSLLKTVKAVEDEATKGTRALEATTEHIRQELAVFCSPEPPAKTSTPEDFIRMTKGITMA 2185

Gg Tn1 2085 DVAKALGDLIGATKAAAGKAGDDPAVYQLKNSAKVMVTNVTSLLKTVKAVEDEATKGTRALEATIEHIRQELAVFSSPVPPAQVSTPEDFIRMTKGITMA 2184

Tn Tn1 2086 DVAKALANLISTTKAAAGKPYDDPSMLQLKSSAKVMVTNVTSLLKTVKAVEDEATKGTRALEATIEHIKQELTVFNGSDAPPRTTAPEEFIRMTKGITMA 2185

Hs Tn2 2087 DVAKALSDLISATKGAASKPVDDPSMYQLKGAAKVMVTNVTSLLKTVKAVEDEATRGTRALEATIECIKQELTVFQSKDVPEKTSSPEESIRMTKGITMA 2186

Gg Tn2 2038 DVAKALSDLIGATKGAASKPADDPSMYQLKGAAKVMVTNVTSLLKTVKAVEDEATRGTRALEATIEYIKQELTVFQSNEVPEKTSSPEESIRMTKGITMA 2137

Tn Tn2 2074 DVAKALAELISATKCAAGKAADDPSMYQLKSAAKVMVTNVTSLLKTVKAVEDEATRGTRALEATIECIKQELALFQSKDPPTKTTTPEEFIRMTKGITTA 2173

Ci Tn-a 2082 DVASALADLINSTKDAAGKSTSDQAMFHLKASAKAMVTNVTSLLKTVKSVEDEAAKGPRAIEQTINSIKQELKSLQSVAGEERRATPEELIQVTKPITNA 2181

Ci Tn-b 2082 DVASALADLINSTKDAAGKSTSDQAMFHLKASAKAMVTNVTSLLKTVKSVEDEAAKGPRAIEQTINSIKQELKSLQSVAGEERRATPEELIQVTKPITNA 2181

*** ** ** :** ** * * :: :** :** *************:*****::* **:* * *:*** : ::**: *::** ** *

Hs Tn1 2186 TAKAVAAGNSCRQEDVIATANLSRRAIADMLRACKEAAYHPEVAPDVRLRALHYGRECANGYLELLDHVLLTLQKPSP-ELKQQLTGHSKRVAGSVTELI 2284

Gg Tn1 2185 TAKAVAAGNSCRQEDVIATANLSRRAIADMLRACKEAAYHPEVSADVRQRALRFGKECADGYLELLEHVLVILQKPTH-ELKQQLAGYSKRVASSVTELI 2283

Tn Tn1 2186 TAKAVAAGNSCRQEDVIATANLSRRAITDMLHSCKQAAYHPEVNKEVRSRALRYGSECASGYLGLLERVLVTIQKPSH-ELKQQLAVDSKRVAGCVTELI 2284

Hs Tn2 2187 TAKAVAAGNSCRQEDVIATANLSRKAVSDMLTACKQASFHPDVSDEVRTRALRFGTECTLGYLDLLEHVLVILQKPTP-ELKQQLAAFSKRVAGAVTELI 2285

Gg Tn2 2138 TAKAVAAGNSCRQEDVIATANLSRKAVADMLTACKQASYHQEVSEEVRERALRFGTECTLGYLELLEHVLLILQKPTP-ELKHQLAAFSKRVASAVTELI 2236

Tn Tn2 2174 TAKAVAAGNSARQEDIIHTANLSRKAMSDMLSTCKQAAYHPDVSEEVKNRALMFGSQCATGYIDLLEHVLLVLQKPSA-EFKQQLAVCSKRVAGAVTELI 2272

Ci Tn-a 2182 TAKAVAAGKFCRQEDMIVCANMGRKAVFDMIHICRASAANTEDPIQQQD-TLKFGSGVAEAYVNLLSNVLATSHQPSNNDLKKNLVPLSKSVATAVSNLV 2280

Ci Tn-b 2182 TAKAVAAGKFCRQEDMIVCANMGRKAVFDMIHICRASAANTEDPIQQQD-TLKFGSGVAEAYVNLLSNVLATSHQPSNNDLKKNLVPLSKSVATAVSNLV 2280

******** :****:* :**: *:*: **: *: :: : : : :* :* : *: ** ** ::*: : *::* ** ** :*: *:

Hs Tn1 2285 QAAEAMKGTEWVDPEDPTVIAENELLGAAAAIEAAAKKLEQLKPRAKPKEADESLNFEEQILEAAKSIAAATSALVKAASAAQRELVAQGKVGAIPANAL 2384

Gg Tn1 2284 QAAEAMKGTEWVDPEDPTVIAENELLGAAAAIEAAAKKLEQLKPRAKPKQADESLDFEEQILEAAKSIAAATSALVKAASAAQRELVAQGKVGVIPANAV 2383

Tn Tn1 2285 QAAEAMKGTEWVDPEDPTVIAENELLGAAAAIEAAAKKLEQLRPRTKPKEADESLNFEEQILEAAKSIAAATSALVKAASAAQRELVAQGKVGAIPANAM 2384

Hs Tn2 2286 QAAEAMKGTEWVDPEDPTVIAETELLGAAASIEAAAKKLEQLKPRAKPKQADETLDFEEQILEAAKSIAAATSALVKSASAAQRELVAQGKVGSIPANAA 2385

Gg Tn2 2237 QSAEAMKGTEWVDPEDPTVIAETELLGAAASIEAAAKKLEQLKPRAKPKQADETLDFEEQILEAAKSIAAATSALVKSASAAQRELVAQGKVGAIPANAA 2336

Tn Tn2 2273 QTAEAMKGSEWVDPEDPTVIAETELLGAAASIEAAAKKLEQLKPRAKPKQADETLNFEEQILEAAKSIAAATSALVKSASAAQRELVAQGKVGLISANAE 2372

Ci Tn-a 2281 RSGESMKGTDWVDPNDPNVIAEQELLAAASSIEAAAKKLAQLRPRKKPKQADESLNFEEQILEAAKSIATATTALVKAASAAQKELVLQGKVGSVPAMRH 2380

Ci Tn-b 2281 RSGESMKGTDWVDPNDPNVIAEQELLAAASSIEAAAKKLAQLRPRKKPKQADESLNFEEQILEAAKSIATATTALVKAASAAQKELVLQGKVGSVPAMRH 2380

:: *:***::**** **:**** *** **::******** **:** ***:***:* *************:**:****:*****:*** ***** : *

Hs Tn1 2385 DDGQWSQGLISAARMVAAATNNLCEAANAAVQGHASQEKLISSAKQVAASTAQLLVACKVKADQDSEAMKRLQAAGNAVKRASDNLVKAAQKAAAFEEQE 2484

Gg Tn1 2384 DDGQWSQGLISAARMVAAATNNLCEAANAAVQGHASEEKLISSAKQVAASTAQLLVACKVKADHDSEAMKRLQAAGNAVKRASDNLVKAAQKAAAFQDHD 2483

Tn Tn1 2385 DDGQWSQGLISAARMVAAATNNLCEAANSAVQGHASEEKLISSAKQVAASTAQLLVACKVKADQDSQTMKRLQAAGNAVKRASDNLVKAAQKAAFEAEDD 2484

Hs Tn2 2386 DDGQWSQGLISAARMVAAATSSLCEAANASVQGHASEEKLISSAKQVAASTAQLLVACKVKADQDSEAMRRLQAAGNAVKRASDNLVRAAQKAAFGKADD 2485

Gg Tn2 2337 DDGQWSQGLISAARMVAAATSNLCEAANASVQGHASEEKLISSAKQVAASTAQLLVACKVKADHDSEAMRRLQAAGNAVKRASDNLVRAAQKAAFGKAED 2436

Tn Tn2 2373 DDGQWSQGLISAARMVAAATSNLCEAANASVQGHASEEKLISSAKQVAASTAQLLVACKVKADQDSEAMRRLQIAGNAVKKASDNLVRAAQNAAFNKADD 2472

Ci Tn-a 2381 DDGQWSQGLISAAQMVARATGNLCEAANQAVQGEASEEKLVTSAKQVASSTAQLLVACKVKADPNSENMKRLQIAGNAVKHASEDLVKAASESANS-DDE 2480

Ci Tn-b 2381 DDGQWSQGLISAAQMVARATGNLCEAANQAVQGEASEEKLVTSAKQVASSTAQLLVACKVKADPNSENMKRLQTAGTAVNKATQMLVESASATFEEQNEE 2479

*************:*** ** ****** :*** **:***::******:************** *: *:*** **:** :*: ** :* : :

Hs Tn1 2485 NETVVVKEKMVGGIAQIIAAQEEMLRKERELEEARKKLAQIRQQQYKFLP SELRDEH 2541

Gg Tn1 2484 -ETVVVKEKMVGGIAQIIAAQEEMLRKERELEEARKKLAMIRQQQYKFLP TELRDEEQN 2541

Tn Tn1 2485 -QAVVVKSKMVGGIAQIIAAQEEMLRKERELVEARKKLAMIRQQQYKFLP SELREDS 2540

Hs Tn2 2486 -DDVVVKTKFVGGIAQIIAAQEEMLKKERELEEARRKLAQIRQQQYKFLP TELREDEG 2542

Gg Tn2 2437 -DDVVVKTKFVGGIAQIIAAQEEMLKKERELEEARKKLAQIRQQQYKFLP TELREDEG* 2494

Tn Tn2 2473 -DNVVVKTKFVGGIAQIIAAQEEMLRKERELEEARKKLAQIRQQQYKFLP SELREDNN 2529

Ci Tn-a 2481 -VEVVINSRLVGGIAQEMMAQEEILRKERELQSARQKLAQIRRMRYKDDS ESD 2531

Ci Tn-b 2480 -PEVELAGGLVSGIAQEMQAMEAILAKEKELKEAQSQLLKIRKKKYEQNQ KK 2531

* : * **** : * * :* **:** *: :* **: :*

**B.**

|  | HsTn1 | Gg Tn1 | Tn Tn1 | Hs Tn2 | Gg Tn2 | Tn Tn2 | Ci Tn-a | Ci Tn-b |
| --- | --- | --- | --- | --- | --- | --- | --- | --- |
| Hs Tn1 |  | 93.0 | 88.6 | 84.9 | 83.5 | 82.8 | 69.9 | 69.4 |
| Gg Tn1 | 88.8 |  | 89.9 | 85.4 | 84.4 | 83.5 | 70.4 | 69.9 |
| Tn Tn1 | 82.3 | 84.5 |  | 84.2 | 83.1 | 82.4 | 69.7 | 69.1 |
| Hs Tn2 | 75.8 | 77.8 | 76.0 |  | 95.6 | 91.8 | 69.9 | 69.3 |
| Gg Tn2 | 74.7 | 77.1 | 75.1 | 94.0 |  | 90.8 | 69.4 | 68.7 |
| Hs Tn2 | 73.5 | 74.9 | 73.8 | 86.8 | 85.8 |  | 69.2 | 68.5 |
| Ci Tn-a | 57.1 | 58.3 | 57.7 | 58.4 | 58.1 | 57.8 |  | 98.5 |
| Ci Tn-b | 56.5 | 57.7 | 57.1 | 57.8 | 57.6 | 57.1 | 98.2 |  |
