# Supplementary material for: Evidence that talin alternative splice variants from Ciona intestinalis have different roles in cell adhesion
Source: BMC Cell Biol. 2006 Dec 6;7:40. doi: 10.1186/1471-2121-7-40 (PMC1702346; doi:10.1186/1471-2121-7-40)
Supplement: Additional File 2 — Alignment of C. intestinalis Talin-a protein sequences. Protein sequences are from the EST sequences listed in Table 1. The alternatively spliced exon sequence is in red. [file 1471-2121-7-40-S2.doc]

**Additional File 2.**

| **GenBank**  **accession**  **number** | **Source** |
| --- | --- |
| BW500911 | adult |
| BW312515 | heart |
| BW261786 | gastrula/neurula |
| BW250883 | tailbud embryo |
| BW229400 | larva |
| BW054944 | blood cells |
| BW170650 | neural complex |
| BP020498 | testis |
| CJ430072 | larva (*M. tectiformis*) |

BW500911 ------------------------------------------------------------

BW312515 ------------------------------------------------------------

BW261786 -------------------HEEILEAAKSIATATTALVKAASAAQKELVLQGKVGSVPAM

BW250883 ------------------------------------------------------------

BW229400 LAQLRPRKKPKQADESLNFEEQILEAAKSIATATTALVKAASAAQKELVLQGKVGSVPAM

BW054944 ------------------------------------------------------------

BW170650 ------------------------------------------------------------

BP020498 ------------------------------------------------------------

CJ430072 ------------------------------------------------------------

BW500911 ------------------------------------------------------------

BW312515 ------------------------------------------------------------

BW261786 RHDDGQWSQGLISAAQMVARATGNLCEAANQAVQGEASEEKLVTSAKQVASSTAQLLVAC

BW250883 ------------------------------------------------------------

BW229400 RHDDGQWSQGLISAAQMVARATGNLCEAANQAVQGEASEEKLVTSAKQVASSTAQLLVAC

BW054944 ------------------------------------------------------------

BW170650 ------------------------------------------------------------

BP020498 ------------------------------------------------------------

CJ430072 RYDDGQWSQGLISAAQTVAGATGNLCEAANQAVQGEASEEKLISSAKQVAASTAQLLVAC

BW500911 ---------------**IAGNAVKHASEDLVKAASESANS-DDEVEVVINSRLVGGIAQEMM**

BW312515 ---------------**IAGNAVKHASEDLVKAASESANS-DDEVEVVINSRLVGGIAQEMM**

BW261786 KVKADPNSENMKRLQ**IAGNAVKHASEDLVKAASESANS-DDEVEVVINSRLVGGIAQEMM**

BW250883 ----------MKRLQ**IAGNAVKHASEDLVKAASESANS-DDEVEVVINSRLVGGIAQEMM**

BW229400 KVKADPNSENMKRLQ**IAGNAVKHASEDLVKAASESANS-DDEVEVVINSRLVGGIAQEMM**

BW054944 ---------------**IAGNAVKHASEDLVKAASESANS-DDEVEVVINSRLVGGIAQEMM**

BW170650 ---------------**----AVKHASEDLVKAASESANS-DDEVEVVINSRLVGGIAQEMM**

BP020498 ---------------**--------------KAASESANS-DDEVEVVINSRLVGGIAQEMM**

CJ430072 **KVKADPNSENMKRLQIAGNAVKHASESLVGAAREAASFQDEEGEVVINERRVGGIAQEMM**

BW500911 **AQEEILRKERELQSARQKLAQIRRMRYKDDSESD**

BW312515 **AQEEILRKERELQSARQKLAQIRRMRYKDDSESD**

BW261786 **AQEEILRKERELQSARQKLAQIRRMRYKDDSESD**

BW250883 **AQEEILRKERELQSARQKLAQIRRMRYKDDSESD**

BW229400 **AQEEILRKERELQSARQKLAQIRRMRYKDDSESD**

BW054944 **AQEEILRKERELQSARQKLAQIRRMRYKDDSESD**

BW170650 **AQEEILRKERELQSARQKLAQIRRMRYKDDSESD**

BP020498 **AQEEILRKERELQSARQKLAQIRRMRYKDDSESD**

CJ430072 **AQEEILRKERELQVAREKLAQIRKWRYKDESESDEE**
