# Supplementary material for: Evidence that talin alternative splice variants from Ciona intestinalis have different roles in cell adhesion
Source: BMC Cell Biol. 2006 Dec 6;7:40. doi: 10.1186/1471-2121-7-40 (PMC1702346; doi:10.1186/1471-2121-7-40)
Supplement: Additional File 3 — Alignment of C. intestinalis Talin-b protein sequences. Protein sequences are from the EST sequences listed in Table 1. The alternatively spliced exon sequence is in red. [file 1471-2121-7-40-S3.doc]

**Additional File 3.**

| **GenBank accession number** | **Source** |
| --- | --- |
| BW056464 | blood cells |
| BW050858 | blood cells |
| BW029542 | blood cells |
| BW029446 | blood cells |
| BW029292 | blood cells |
| BW050224 | blood cells |
| BW482633 | adult |
| BW522112 | gastrula (*C. savignyi*) |

BW056464 --------------------------------------------GEEKLVTSAKQVASST

BW050858 ------------------------------------------------------------

BW029542 VGSVPAMRHDDGQWSQGLISAAQMVARATGNLCEAANQAVQGEASEEKLVTSAKQVASST

BW029446 -------------------------------------QAVQGEASEEKLVTSAKQVASST

BW029292 -----------------------------------------RHEGEEKLVTSAKQVASST

BW050224 ------------------------------------------------------------

BW482633 ------------------------------------------------------------

BW522112 VGSIAAMRHDDGQWSQGLISAAQMVARATGNLCEAANQAVQGEASEEKLVTSAKQVASST

BW056464 AQLLVACKVKADPNSENMKRLQ**TAGTAVNKATQMLVESASATFEEQNEEPEVELAGGLVS**

BW050858 -----------------RHEVQ**TAGTAVNKATQMLVESASATFEEQNEEPEVELAGGLVS**

BW029542 AQLLVACKVKADPNSENMKRLQ**TAGTAVNKATQMLVESASATFEEQNEEPEVELAGGLVS**

BW029446 AQLLVACKVKADPNSENMKRLQ**TAGTAVNKATQMLVESASATFEEQNEEPEVELAGGLVS**

BW029292 AQLLVACKVKADPNSENMKRLQ**TAGTAVNKATQMLVESASATFEEQNEEPEVELAGGLVS**

BW050224 ----------------------**--------ATQMLVESASATFEEQNEEPEVELAGGLVS**

BW482633 ----------------------**--------------------------------------**

BW522112 AQLLVACKVKADPNSENMKRLQ**SAGTAVNRATQMLVESASASFEVQ--------------**

BW056464 **GIAQEMQAMEAILAKEKELKEAQSQLLKIRKKKYEQNQKK**

BW050858 **GIAQEMQAMEAILAKEKELKEAQSQLLKIRKKKYEQNQKK**

BW029542 **GIAQEMQAMEAILAKEKELKEAQSQLLKIRKKKYEQNQKK**

BW029446 **GIAQEMQAMEAILAKEKELKEAQSQLLKIRKKKYEQNQKK**

BW029292 **GIAQEMQAMEAILAKEKELKEAQSQLLKIRKKKYEQNQKK**

BW050224 **GIAQEMQAMEAILAKEKELKEAQSQLLKIRKKKYEQNQKK**

BW482633 **-------AMEAILAKEKELKEAQSQLLKIRKKKYEQNQKK**

BW522112 **----------------------------------------**
